# A diversified and segregated mRNA spliced-leader system in the parasitic Perkinsozoa

**DOI:** 10.1098/rsob.220126

**Published:** 2022-08-24

**Authors:** Elisabet Alacid, Nicholas A. T. Irwin, Vanessa Smilansky, David S. Milner, Estelle S. Kilias, Guy Leonard, Thomas A. Richards

**Affiliations:** ^1^ Department of Zoology, University of Oxford, Oxford, Oxfordshire OX1 3SZ, UK; ^2^ Merton College, University of Oxford, Oxford, Oxfordshire OX1 4JD, UK; ^3^ Living Systems Institute, University of Exeter, Exeter, Devon EX4 4QD, UK

**Keywords:** gene expression, mRNA processing, *trans*-splicing, spliced-leader RNA, parasite, alveolata

## Abstract

Spliced-leader *trans*-splicing (SLTS) has been described in distantly related eukaryotes and acts to mark mRNAs with a short 5′ exon, giving different mRNAs identical 5′ sequence-signatures. The function of these systems is obscure. Perkinsozoa encompasses a diversity of parasitic protists that infect bivalves, toxic-tide dinoflagellates, fish and frog tadpoles. Here, we report considerable sequence variation in the SLTS-system across the Perkinsozoa and find that multiple variant SLTS-systems are encoded in parallel in the ecologically important Perkinsozoa parasite *Parvilucifera sinerae*. These results demonstrate that the transcriptome of *P. sinerae* is segregated based on the addition of different spliced-leader (SL) exons. This segregation marks different gene categories, suggesting that SL-segregation relates to functional differentiation of the transcriptome. By contrast, both sets of gene categories are present in the single SL-transcript type sampled from *Maranthos,* implying that the SL-segregation of the *Parvilucifera* transcriptome is a recent evolutionary innovation*.* Furthermore, we show that the SLTS-system marks a subsection of the transcriptome with increased mRNA abundance and includes genes that encode the spliceosome system necessary for SLTS-function. Collectively, these data provide a picture of how the SLTS-systems can vary within a major evolutionary group and identify how additional transcriptional-complexity can be achieved through SL-segregation.

## Graphical abstract

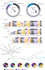

## 1. Introduction

Spliced-leader *trans*-splicing (SLTS) has been described in many eukaryotic organisms, including paraphyletic groups from diverse evolutionary phyla [1–3]. SLTS involves the transfer of a short RNA fragment, the spliced-leader (SL), from the 5′ end of a non-coding RNA, the SL RNA, on to pre-mRNA molecules, a process catalysed by the spliceosome. As a result, diverse mRNAs acquire a common 5′ sequence. SL-RNAs carry the donor splice site that divides the short molecule into two distinct parts: the 5′ end is the SL sequence or SL exon (SLe), which is transferred to the pre-mRNA along with the 5′-cap structure; the 3′ portion or the SL intron (SLi) contains the Sm-binding site for Sm proteins and forms a Y-branched product with the pre-mRNA outron, which is degraded after the SLe-sequences have been spliced onto the mRNA [2,4]. A variety of purposes for SL *trans*-splicing have been proposed, most of them involving processing, regulation or translation of the mRNA [2,5]. Yet, the functional and evolutionary reason to maintain these systems remains a quandary.

Within the protist group Alveolata, SLTS was first discovered in dinoflagellates [6], a lineage that includes species which significantly contribute to marine primary production and growth of corals through photo-symbiosis, and can form harmful algal blooms [7]. Throughout all major dinoflagellate orders, the nuclear-encoded mRNAs coding for diverse proteins are *trans*-spliced with the addition of a 22-nt conserved SLe with evidence of sequence degeneration only in the first nucleotide position [6,8,9]. The SLe is provided by SL-RNA molecules of 50–60 nt, with a conserved Sm-binding motif located in the exon rather than within the intron region. Later, the discovery of SLTS in the shellfish parasite *Perkinsus marinus* identified a homologous SLTS-system operating in the Perkinsozoa, the sister lineage of the dinoflagellates [10–13]. Currently, the *Perkinsus* genus is the only taxon for which there is evidence of a SLTS-system within the Perkinsozoa lineage. These studies reported that *P. marinus* has a 22nt (ACCGTAGCCATCTTGGCTCAAG) SLe and a shorter 21nt (ACCGTAGCCATCTGGCTCAAG) SLe provided by a SL-RNA precursor of 80–83 nt. These two SLe-sequences vary slightly from that of dinoflagellates (DCCGTAGCCATTTTGGCTCAAG, D = A, G, T), specifically in the middle of the sequence, where the TTTT motif in dinoflagellates differs from the TCTT and TCT found in *P. marinus*. SLTS seems to be absent in other alveolates sampled, specifically the apicomplexans and the ciliates, suggesting the system has been acquired early in the evolution of Dinozoa [14,15], or even earlier but then lost in the ancestor of the ciliates and separately in the ancestor of the apicomplexans.

The phylum Perkinsozoa encompasses a diversity of widespread aquatic protistan parasites with important ecological and economic implications [16]. Representative groups include infectious agents of commercial bivalve species [17], toxic-bloom forming dinoflagellates [18–21], farmed and wild fish [22–24] and Anura tadpoles [25,26]. In order to better understand the evolution and function of the SL *trans*-splicing mechanism in this important parasitic group, there is the need to sample taxa from across this taxonomic group.

Here, we explore the evolutionary diversification of the Perkinsozoa SLTS-system from genomic and transcriptomic data from two parasites of toxic-bloom forming dinoflagellates, *Parvilucifera sinerae* and *Maranthos nigrum*, as well as a meta-genomic survey of tadpole tissues with Severe Perkinsea Infections (SPI). These dinoflagellate parasite taxa were selected for further study because the host–pathogen interaction is in stable culture and available small subunit ribosomal RNA (SSU rRNA) gene sequences are divergent and/or subject to fast rates of evolutionary diversification, suggesting the taxa represent useful organisms for Perkinsozoa wide comparisons of SLTS variation. Our results show a diversified SL system in parasitic Perkinsozoa and identify how increased transcriptional-complexity can be achieved through SL segregation of the transcriptome. This study provides further understanding of how the SLTS-systems can vary within a major taxonomic group and identifies several functional characteristics of the SLTS-systems in Perkinsozoa relating to differential transcript abundance patterns and segregation of the transcriptome into functional groupings by addition of variant SL sequences.

## 2. Results and discussion

### 2.1. Diversification of the Perkinsozoa spliced-leader *trans*-splicing system

**Figure 1.**
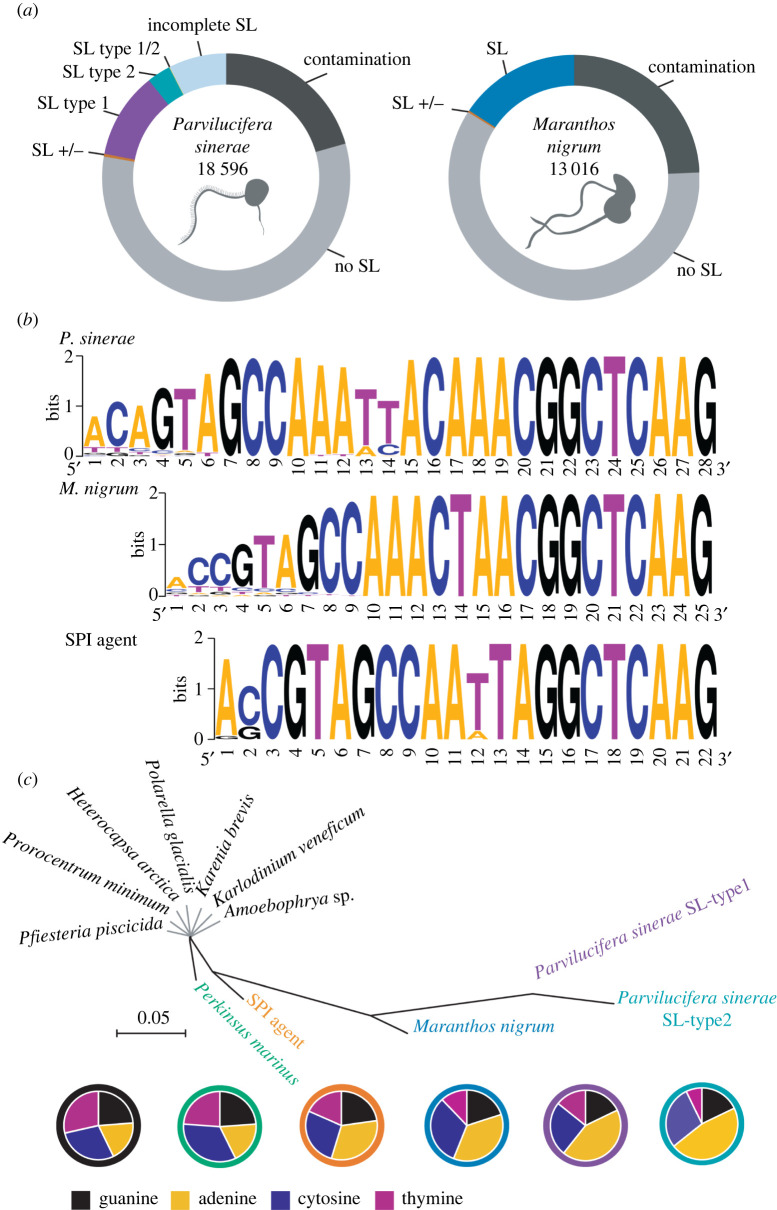
Evolution of the SLTS-system in the Perkinsozoa. (*a*) Percentage of non-SL and SL-bearing transcripts in the *P. sinerae* and *M. nigrum* zoospore transcriptomes. In *P. sinerae*, we obtained 23 232 transcripts. After removing 4646 transcripts as putative sources of contamination, we identified 18 596 transcripts (representing a BUSCO score of 76.7% ‘completeness’). A total of 4935 transcripts contained evidence of an SL-sequence (26.54% of the total transcriptome) whereas 12 880 transcripts did not (no SL). Two types of SL-sequences were identified: the SL sequence of 2541 transcripts were long enough (15nt) to be classified as SL-type-1, while the SL sequence of 691 transcripts were also long enough (15 nt) to be classified as SL-type-2. Five transcripts were found to have variant (both) SL-types (SL-type-1/2), 81 transcript isoforms were shared between non-SL and SL-containing transcripts (SL +/−) and 1703 *Parvilucifera* transcripts contained an incomplete SL-sequence, i.e. were shorter than 15nt (and so classified as ‘Incomplete SL’), so they could not be attributed to either SL-type. In the case of *M. nigrum*, we obtained 17 108 transcripts, which after removing potential contamination, we retained 13 016 transcripts. The BUSCO score for transcriptome completeness was 41.5%. From the total number of transcripts, 2652 contained evidence of a splice-sequence (SL), representing 20.4% of the total transcriptome, whereas 10 024 did not (No SL). Forty-three transcripts were shared between non-SL and SL-containing transcripts (SL +/−). See also electronic supplementary material, figure S1 and S2. Note that these numbers are likely to be an underestimate of the frequency of the occurrence of the spliced-leader sequence modifications because a percentage of transcripts might have an incomplete 5′ end, the identification of which requires full-length or nearly full-length transcripts. Some 5′ ends are possibly missing due to technical limitations of the sequence sampling approach. Analysis of partial SLs (electronic supplementary material, figure S1) and comparison between SL-PCR and Illumina RNA-seq libraries (electronic supplementary material, figure S3) suggests that the estimate error of SL-transcripts misclassified as non-SL transcripts due to incomplete 5′ ends is approximately 10% for both species (see 'Results and discussion' for more information). (*b*) Sequence logos [27] generated from the alignment of the SLe-sequences from each Perkinsozoa. The SLe length is shown and the overall height of the stack indicates the sequence conservation at each position, while the height of symbols within the stack indicates the relative frequency of each of the four nucleotides at each position. (*c*) Unrooted parsimony tree (gaps as a fifth character state) of SLe-sequences showing the percentage of nucleotide content represented as pie charts. SLe-sequences used for *P. sinerae*, *M. nigrum* and SPI agent have been determined in the present study. SLe-sequences used from *Perkinsus* and dinoflagellates have been obtained previously [6,9,11,12,28]. Grey lines are for labelling. The scale bar denotes the number of character changes across the given branch length. Each pie chart is encircled by the same colour as the species font colour: Dinoflagellates (black), *Perkinsus marinus* (green), SPI agent (orange), *M. nigrum* (blue), *P. sinerae* SL-type 1 (purple) and *P. sinerae* SL-type 2 (turquoise).

Transcriptome analysis of the zoospore development stage of the dinoflagellate parasite *Parvilucifera sinerae* identified 4935 distinct transcripts (26.54% of the total transcriptome) possessing an identifiable SL sequence (figure 1*a,b*). Many of the assembled transcripts were missing the 5′ end of the SL and only 12 transcripts (0.24%) had the complete 28 nt of the SL sequence. A comparison of the recovery of the 5′ end of the SL sequence recovery is detailed in electronic supplementary material, figures S1a and S2a and demonstrates that the rest of these transcripts contained a partial SL missing a subsection of the 5′ end of the transcript. Importantly, 66% of the *Parvilucifera* transcripts had a SL sequence of at least 15 nucleotides (electronic supplementary material, figure S1a) allowing us to classify them as type 1 or type 2 SL sequence. Spliced-leader type-1 (SL-type-1) was present in 2541 transcripts (51.4% of SL-bearing transcripts), while SL-type-2 was present in 691 transcripts (14%) (figure 1*a*). The rest of the SL-containing transcripts had an incomplete SL sequence (1703, 34.5% of SL-containing transcripts), where the nucleotides missing at the 5′ end of the SL sequence included the variable region, and thus we could only identify 14 nt or less of the SL sequence. As such we could not assign these ‘incomplete SL’ sequences to either SL-type-1 or SL-type-2. Such sequences are referred to as ‘incomplete’ (figure 1*a*, light blue colour; electronic supplementary material, figure S1a). The two SLe types were not present at any of the 5′UTR regions in the corresponding genes identified from the genome sequence assembly (electronic supplementary material, figure S2*b*), demonstrating that this sequence is an SLe *trans*-spliced post-transcriptionally onto a subset of the *Parvilucifera sinerae* zoospore mRNA.

Within the *Maranthos nigrum* zoospore transcriptome, 2652 identified transcripts (20.4% of the total transcriptome) contained a single 25 nt 5′ SL sequence (figure 1*a*,*b*; electronic supplementary material, figure, S1*b* and S2*c*). From these recovered SL-containing transcripts, 36 of them (1.36%) presented a complete sequence and the rest contained partial SL sequences at the 5′ end (electronic supplementary material, figure S1*b*). These short sequences were not present at any of the 5′ UTR regions in the corresponding genes identified from the genome sequence assembly (electronic supplementary material, figure S2*d*), again confirming this sequence was *trans*-spliced after transcription.

It is important to note the high number of partial SL sequences recovered for both parasite species (electronic supplementary material, figure S1) suggests we are likely to be underestimating the occurrence of transcripts that are SL *trans*-spliced. Missing nucleotides at the 5′ end of transcripts is expected in RNA-seq data due to technical/methodological limitations in mRNA sequence sampling, such as RNA degradation and low coverage sequencing of transcript ends compared to the core transcript body [29]. We have therefore analysed the abundance distribution of transcripts with missing nucleotides at the 5′ end of the SL sequences (electronic supplementary material, figure S1). The graph shows that the data represents a normal distribution with a peak of transcripts with an SL sequence of at least 16 nucleotides, and where over 90% of transcripts with evidence of an SL had at least the last 10 nucleotides of the SLe conserved at the 3′ end region for both species. These data suggest that the estimated error of transcripts missing the 5′ end due to technical/methodological limitations is below 10% assuming failure to recover nucleotide positions from the 5′ is a normally distributed phenomenon.

To further check for this source of error, we performed a SL PCR-based experiment to compare the number of transcripts that overlap between the multiplex SL-PCR libraries and the two pools of transcripts, SL-bearing transcripts (all-SL zoospore) and transcripts without (non-SL zoospore) from the Illumina RNA-seq libraries (electronic supplementary material, figure S3). Although the SL-PCR amplification was performed in a sample including all life cycle stages, and thus, not all the SL-transcripts from the zoospore dataset are likely to be recovered in the SL-PCR amplified library, the percentage of SL-containing transcripts from the zoospore stage that are identified by the SL-PCR method was approximately 40% and approximately 60% in *M. nigrum* and *P. sinerae*, respectively (electronic supplementary material, figure S3a and c). However, the number of transcripts overlapping between the SL-PCR library and non-SL containing transcripts from the Illumina RNA-seq library was 7% and 13% in *M. nigrum* and *P. sinerae*, respectively (electronic supplementary material, figure S3b and d). Again, this analysis suggests that the estimated error of putative SL-bearing transcripts misclassified as non-SL transcripts in the Illumina RNA-seq dataset is approximately 10%. It is important to note the high overlap between the multiplex SL-type-1-PCR and SL-type-2-PCR libraries demonstrates that SL-based PCR approaches prevented us from discriminating between variant SLs due to low primer specificity leading to cross-amplification. Thus, we found that this method is not useful in the case of Perkinsozoa for distinguishing variant SL forms as is also the case for other organisms with different SL types which possess a high degree of sequence similarity [30].

**Figure 2.**
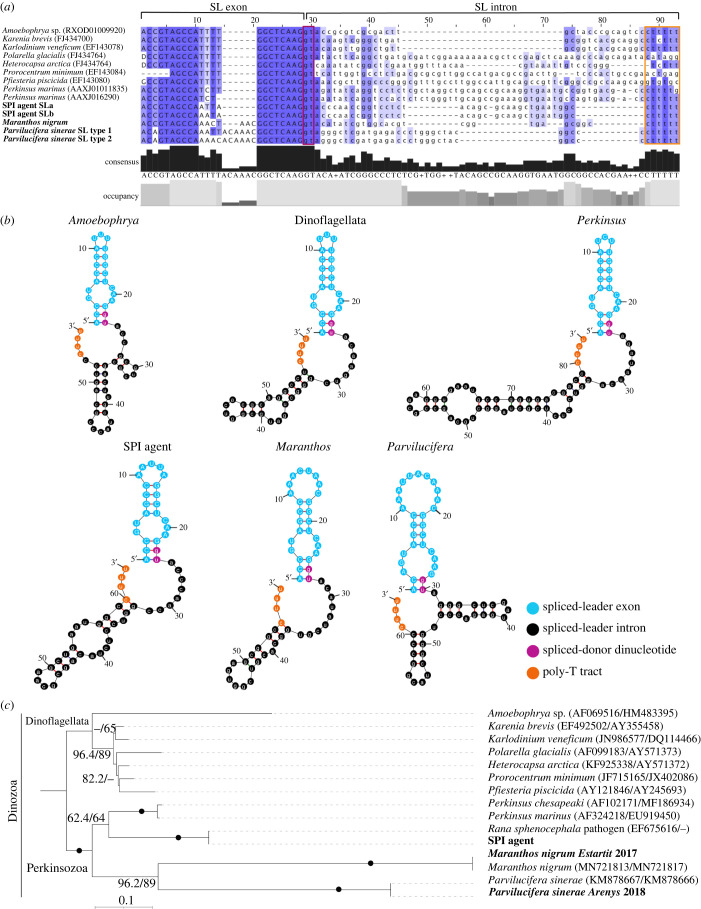
Conservation of spliced-leader sequences and their RNA structures in the Dinozoa clade. (*a*) Alignment of representative sequences of the SLe–SLi ORFs of Dinozoa representatives based on their secondary structure. The bold labels of the SPI agent, *M. nigrum* and *P. sinerae* indicate sequences identified in this study (see also electronic supplementary material, figure S4). The *P. marinus* [11] and dinoflagellate [6,9,28] sequences include their accession numbers within parenthesis. Note that *P. minimum* contains a partial SLe-sequence due to the primer used in the study [6]. Gaps introduced in the sequence alignment are shown as ‘–’. D = T, A or G. SLe are shown in uppercase letters whereas the intron is in lowercase. Purple shaded areas represent the percentage of similarity in the sequences. Splice-donor dinucleotide is boxed in pink and the poly-T tract is boxed in orange. Jalview v. 2.11.1.6 [31] was used for multiple sequence alignment editing and visualization. (*b*) Predicted secondary structures of SLe–SLi ORFs sequences using Mfold webserver [32] (http://www.unafold.org/mfold). *Karenia brevis* SLe–SLi-sequence is used as a dinoflagellate representative to show the conserved secondary structure in the group. Key regions of the SL-RNA gene are displayed in different colours: SLe (blue); SLi (black); splice-donor dinucleotide (pink); poly-T tract (orange). (*c*) Maximum-likelihood phylogenetic tree inferred in IQ-TREE with a partitioned model of GTR + F + I + G4 and TN + F + R3 for the concatenated SSU and LSU rRNA gene sequences of Dinozoa. Values at branches correspond to ultra fast bootsrap analyses and SH-aLRT supports (1000 replicates). Support values are summarized by black circles when both are greater than or equal to 98%/90%. Sequences obtained in this study are again labelled with bold names.

To identify the full-length SL-RNA intron (SLi) and exon (SLe) sequences of the identified SLs, we used the SLe-sequences as a query to perform BLASTn-short similarity searches of our *de novo P. sinerae* genome assembly to identify candidate SLe–SLi open reading frames (ORFs) (figure 2; electronic supplementary material, figure S4). For *P. sinerae,* we recovered evidence for three separate ORFs encoding SL-type-1 and one ORF encoding SL-type-2 (figure 2*a*; electronic supplementary material, figure S4*a*). This process also recovered five additional candidate SLe–SLi ORFs with minor sequence variations compared to the two described in figure 2*a* (electronic supplementary material, figures S1*a* and S4*a*). Currently, we have no evidence of functional-splicing for these additional variants.

For *M. nigrum*, using the same process, we confirmed the SLi- and SLe-sequence of the SL identified from transcriptome sequencing and identified evidence for 25 additional SLe–SLi ORFs with minor sequence variations from the *de novo* genome sequence (electronic supplementary material, figure S4*b*). Collectively, these data suggest that the SLe–SLi ORFs have undergone considerable ORF duplication in both *M. nigrum* and *P. sinerae*. Furthermore, these results also suggest that additional variant SL-systems are either operating in both these protists, but are currently undetected in the two zoospore-targeted transcriptome sequencing library sets produced, or that the SLe–SLi ORF has been subject to extensive ‘pseudogenization’.

For the SPI agent, only meta-genomic sequence data were recovered; therefore, we used the SLe-sequences identified from a range of Dinozoa (figure 2*a*) to conduct BLASTn short-sequence similarity searches in the two SPI meta-genomic datasets. This process identified a candidate SLe of 22 nucleotides in length (figure 1*b*) allowing us to identify putative SLe–SLi ORFs (figure 2*a*; electronic supplementary material, figure S4*c*). Specifically, this ORF was found as tandem repeats located close to the 5S ribosomal RNA gene (electronic supplementary material, figure S5) in both independently sampled meta-genomes (KNA03_01 and KNA_DNA). The candidate SLe–SLi ORF was in the same equivalent position within the genome assembly contigs as the identified dinoflagellate SLe–SLi ORF [9]. Indeed, an equivalent SLe–SLi ORF location has been reported in other species [33–36]. This process allowed us to identify candidate SLe–SLi ORFs for the SPI agent (figure 2*a*; electronic supplementary material, figure S4*c*). However, the lack of transcriptome data prevents confirmation that this sequence was *trans*-spliced post-transcriptionally onto mature mRNA transcripts. Furthermore, the absence of transcriptome data and incomplete nature of the genome sequence survey means that the diversity of SPI SL-systems is likely to be underestimated here. Replete transcriptome and genome sequencing are therefore necessary to confirm this result but are currently not possible as we cannot raise a stable culture of the SPI agent, despite many attempts from isolates of diverse geographic provenance.

By cross-referencing to genomic data in all the SLe–SLi ORFs sampled here we were able to identify the poly-T tract marking the termination element of the SLe–SLi RNA (figure 2*a*; electronic supplementary material, figure S4) evident in the three Perkinsozoa investigated here and as reported for dinoflagellates and *Perkinsus* [9,11,12]. These results suggest we have identified complete SLe–SLi ORFs.

### 2.2. Variation in Perkinsozoa spliced-leader *trans*-splicing system

Previously reported SL RNA exon/intron ORFs range from 45 to 150 nt depending on the organism, with the SLe usually 16–51 nt [2,3,37]. The Perkinsozoa SL RNA intron/exon candidate ORFs ranged from 66 nucleotides in length (including the poly-T tract) in the SPI agent (*n* = 19), 60–61 nt in *M. nigrum* (*n* = 25)*,* and 67–68 nt in *P. sinerae* (*n* = 9) (electronic supplementary material, figure S4). For comparison, dinoflagellate RNA exon/intron ORFs range from 50 to 60 nt [6,9]. SLs in Perkinsozoa had greater sequence and secondary structure variation between species relative to sampled dinoflagellates (figure 2*a*,*b*). While dinoflagellates, *Perkinsus* and the SPI agent have SLs with a conserved length of 21–22 nucleotides, *M. nigrum* and *P. sinerae* SLe-sequences are composed of 25 and 28 nucleotides, respectively (figure 1*b*).

Previous studies of SLTS-systems across a number of distinct eukaryotic clades have shown a high degree of sequence conservation within members of the same phylum, but a low degree of conservation between the SLTS-systems from different phyla [1,2,37]. These studies have also indicated that the SLe length has a higher level of conservation than the sequence composition within any given group of phyla. Furthermore, the SLe region shows a higher level of sequence conservation than the SLi-region, with a conserved 3′ where SLe–SLi cleavage occurs and the SLe-sequence is incorporated onto mRNAs. This pattern of conservation is also present across the Dinozoa SLe–SLi ORFs, with patterns of conservation present in the 3′ region of the SLe and regions of conserved similarity within the 5′- and 3′-end intronic regions (figure 2*a*).

Regarding the 5′ ends, the SLe-sequences presented a consensus ACCGTAGCCA pattern in Perkinsozoa, with one nucleotide variation in *P. sinerae* (figure 2*a*). We note that all SLe forms identified shared the last eight nucleotides at the 3′ end and the same terminal nucleotide triplet; AAG (figures 1*b* and 2*a*). The cleavage site between the exon and the intron (GGTA motif) is conserved in the Perkinsozoa (figure 2*a*), from which the first nucleotide G is the last nucleotide of the exon, and the other three nucleotides (GTA) are at the beginning of the intronic region. The intron starts with the 5′ donor splice site GT, where the nucleophilic attack occurs during splicing to release the intron as a Y-branched product while combining the SLe with the first exon of the mRNA transcripts. At the 3′ end of the intronic region, the poly-T tract shows a pattern of CT_5_A in the SPI agent and CT_4-6_G/C in *M. nigrum* and *P. sinerae* at the same location (figure 2*a*; electronic supplementary material, figure S4); similar to the CT_4-7_G/A sequences previously reported for dinoflagellates and *Perkinsus* [9,11,12].

Conserved Sm binding site sequence are generally located in the SLi, with RAT_4–6_GR in the kinetoplastids [38] and freshwater planarians [39], RAT_4_CGG in *Hydra* [35], AGCT_3_GG in *Ciona* [40], AGCT_4_CT_3_GG in *Schistosoma* [41] and AAYTYTGA in the Rotifera [42]. In dinoflagellates, however, no Sm-binding site was found in the SLi-sequences identified, instead, a common Sm variant (AT_4_GG) was detected within the SLe, which has been proposed as the Sm-binding site [6]. We could not identify the classical Sm-binding site composed of a T-rich element in either the SLe- or SLi-sequence of the Perkinsozoa. Notably, the corresponding AT_4–6_G regions found in the dinoflagellate SLe have undergone sequence variation across the Perkinsozoa where thymine content (ranging from 24% to 7%) has largely been replaced with adenines (19-to-46%) (figure 1*c*). Thus, these results suggest (i) the SL-RNA structure is more important than specific sequence motifs in binding *trans*-splicing factors and for splice site recognition and/or (ii) that components of the spliceosome have diverged in the Perkinsozoa resulting in alternative Sm-binding sites and recognition motifs.

The consensus SL-RNA exon/intron ORF sequences for the Perkinsozoa and the dinoflagellates [6,9,11,28] were used to predict their putative secondary structures [1,9,11,12] (figure 2*b*). Due to the differences in the intronic region, we set out to investigate if the predicted secondary structures, to which the spliceosome and small nuclear ribonucleoproteins (snRNPs) attach to perform splicing, are conserved. We found that the predicted secondary structures across the Dinozoa showed variation, having two or three stem-loops and a branch-point in the SL-RNA molecule. The length of the loops and the stems are different across the species sampled, conferring variant shapes to the SL-RNA structures (figure 2*b*). Indeed, whereas dinoflagellates, *Perkinsus*, the SPI agent and *Maranthos* present two stem-loops, *Amoebophrya* and *Parvilucifera* have SL-RNA structures which are composed of three closing helices, a structure that is conserved in most eukaryotic SL-systems [37]. However, in the case of the Dinozoa representatives sampled (figure 2*a*,*b*), the RNA structures identified clearly indicate a structurally conserved 3′-splice donor GU dinucleotide positioned at the exon/intron boundary, delineating an AU base-pairing interaction in the first stem-loop (coloured in purple-blue in figure 2*b*). The structures also identify the position of the conserved GC forming a pair of closing bases within most of the stem-loops of the intron (figure 2*b*). These data identify key structurally conserved SL-elements across the Dinozoa systems sampled.

Taken together, these results demonstrate how the SL-system varies across the Dinozoa, providing a picture of how such systems can vary within a major evolutionary group. While the sampled dinoflagellates with SLe-sequences show a higher degree of sequence conservation, the SLe-sequences and secondary structure of the Perkinsozoa are more variable. The high similarity between *Perkinsus* and dinoflagellates regarding SLe length and nucleotide composition, suggests that the evolution of longer SLe forms encompassing adenine-enriched insertions seen in *M. nigrum* and *P. sinerae* occurred during the evolution of Perkinsozoa, leading to the increased diversification of SL-types (figures 1 and 2). This pattern of SL-sequence variation is consistent with the branch-lengths observed for the concatenated small subunit (SSU) and large subunit (LSU) rRNA gene phylogenies (figure 2*c*), in agreement with previous phylogenetic analysis [16,18,19,21] where the Perkinsozoa, specifically *M. nigrum* and *P. sinerae*, demonstrate divergent patterns of rRNA sequence variation. We note here that the SSU/LSU rRNA gene phylogeny is shown primarily to contrast the patterns of rRNA sequence variation with variation in the SLTS-system and note that the Perkinsozoa phylogenetic tree topology is subject to further revision given additional multi-gene phylogenetic analyses.

### 2.3. *Parvilucifera sinerae* variant spliced-leaders mark discrete transcriptome repertoires

Why certain genes are *trans*-spliced while others are not is an outstanding question. The systematic study of SLTS-systems across a diversity of eukaryotic organisms has led to a range of different proposals regarding the role of these mechanisms. Proposed functions include, for example, processing of polycistronic pre-mRNAs generated from eukaryotic operons, stabilizing mRNAs, removal of regulatory elements from the outron (5′ UTR sanitization), alteration of protein subcellular localization, inclusion/exclusion of ORFs or other regulatory elements from the 5′ end of transcripts, proteome diversification by translation of alternative ORFs, cDNA recycling, enhancement of translation of *trans*-spliced transcripts by providing a hypermethylated 5′ cap and/or marking transcripts for increased mRNA abundance via an unknown mechanism [2,3,43–45].

**Figure 3.**
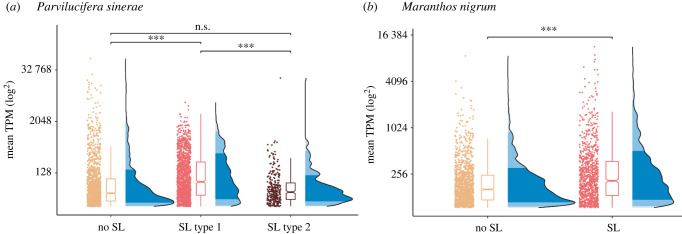
Read mapping frequency analysis of relative transcript abundance of SL trans-spliced transcripts and non-SL transcripts. Read mapping frequency is normalized as transcripts per million (TPM). Box plots summarize the median and upper quartile values along with half-violin plots to show the probability density of the data for each group of transcripts. (*a*) *Parvilucifera sinerae* TPM distribution data in the zoospore transcriptome for each of the three groups: transcripts without SL (yellow), transcripts with SL-type-1 (red) and transcripts with SL-type-2 (brown). Statistical significance between pairwise comparisons using *X*^2^ Kruskal−Wallis test (*p* = 1.62 × 10^−39^; *n*_obs_ = 3195). (*b*) Read mapping frequency of transcripts without SL (yellow) and SL-bearing transcripts (red) in *Maranthos nigrum* zoospore transcriptome. Statistical significance between the read mapping frequency (TPM) of the two groups of transcripts using Mann–Whitney test (*p* = 3.54 × 10^−18^, *n*_obs_ = 2312). ***p-0.001; n.s.; not significant.

To explore the idea that the SLe presence/absence on mRNA may correlate with transcript abundance, we used read mapping frequency analysis to identify the relative abundance patterns of transcripts with SLe-sequences compared to transcripts without this character. These data demonstrated that the SL-type-1 marked transcripts in *P. sinerae* and the SL-marked transcripts in *M. nigrum* showed a statistically significant increase in relative read mapping, demonstrating an increased relative transcript abundance for transcripts with these splice leaders (figure 3*a*,*b*). However, there were no statistical differences between *P. sinerae* SL-type-2 and non-SL-bearing transcripts (figure 3*a*). These results are consistent with the idea that the SL-system is associated with transcripts either being blocked from mRNA breakdown (extending the half-life of the mRNA) and/or that splice-leader modification is associated with a process where a subset of genes is marked for increased transcription, perhaps arising from co-regulation associated with functional classes of transcripts or transcription factors. However, these results do not identify a mechanistic link between the presence of an SL and increased transcript abundance.

An additional leading idea proposed for the role of the SL-system is that certain subsets of functionally related transcripts may be marked by a given SLe whereas others are not, i.e. the transcriptome is segregated by SLs based on categories of protein function. Several studies have explored if SLTS-systems are associated with specific gene categories or associated with function ontologies [1,6,43,46,47]. Most of these studies have demonstrated that although some gene ontological categories were over-represented in some species, there is no conclusive evidence associating the SLTS-mechanism with a specific biological process or functional category [1,6,46,47]. These studies were largely based on the analysis of SL-containing transcripts in isolation and do not compare gene categories or functions associated with transcripts marked by a splice-leader sequence relative to non-SL transcripts across the total transcriptome. However, we note that in *Ciona intestinalis*, where between 50 and 58% of genes are SL *trans*-spliced, different gene ontology categories were found over-represented in highly expressed *trans*-spliced monocistronic genes versus non-*trans*-spliced genes [43], suggesting that genes with different structure, regulation and function are differentially impacted by *trans*-splicing versus conventional expression.

**Figure 4.**
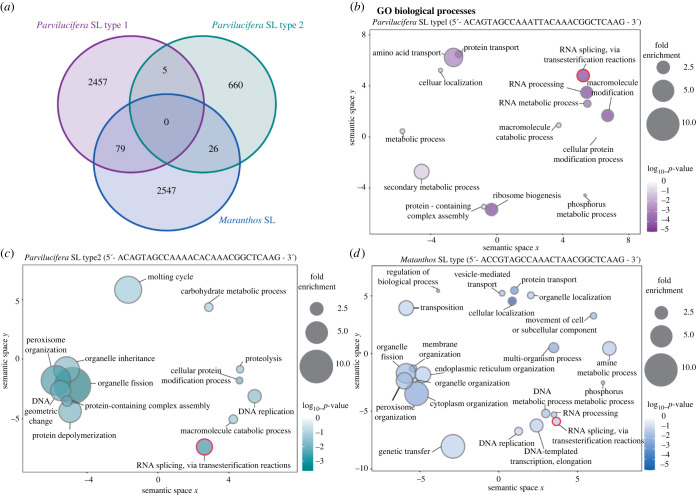
The comparison of shared transcripts and GO-enriched biological processes of SLe-bearing transcripts in *P. sineare* and *M. nigrum* zoospores transcriptomes. (*a*) Number of SL-bearing transcripts shared between the three SL-types identified in *P. sinerae* and *M. nigrum*. Note that there are 0 transcripts/isoforms shared between all three datasets. Biological processes enriched in SL-type-1 (*b*) and SL-type-2 (*c*) marked transcripts in *P. sinerae* in comparison to the total transcriptome (see also electronic supplementary material, table S1). (*d*) Biological processes enriched in SL-transcripts in *M. nigrum* in comparison to the wider transcriptome. Animal-specific GO-enriched terms (see electronic supplementary material, table S1) were excluded from the graph. The size of the circles represents the fold enrichment and the colour intensity represents the *p*-value (log10- see scatterplot key). Note that GO-enriched terms shared between (*b*), (*c*) and (*d*) are encircled in red.

To investigate the potential function of SLTS in Perkinsozoa parasites we examined whether gene ontology terms were enriched in the SL-containing transcripts from *M. nigrum* and *P. sinerae* relative to their total transcriptomes. We argue that *P. sinerae* is a particularly good system for such analyses as it has three groups of transcripts: transcripts with SL-type-1, transcripts with SL-type-2 and transcripts with no detectable SLe-sequence (figure 1*a*). Firstly, it is important to note that we only identified 43 transcript species (0.33%) in *M. nigrum* and 81 transcript species (0.44%) in *P. sinerae* that contained isoforms both with and without an SL (figure 1*a*). Furthermore, for *P. sinerae,* we only identified five (0.03%) transcript species with isoforms with either SL-type-1 and SL-type-2 (figures 1*a* and 4*a*). These data demonstrate that the transcriptome of these two parasites is highly segregated by SL-type in the zoospore phase of the Perkinsozoa life cycle.

Using gene ontology enrichment analysis of subsections of the transcriptome defined by the different SLe-sequences, we sought to test the hypothesis that SL systems mark discrete gene repertoires relating to functional categories. Our analyses demonstrate that the two different SL types found in the *P. sinerae* zoospore transcriptome are enriched for different biological processes, cellular components and molecular functions (figure 4*b–d*; electronic supplementary material, table S1). *Parvilucifera* SL-type-1 is enriched for biological processes related to RNA processing (GO:0006396, *p* < 0.0001), ribosome biogenesis (GO:0042254, *p* < 0.0001), several metabolic processes and transport (figure 4*b*), and for molecular functions related to nucleic acid and peptide binding, and catalytic and transporter activities (see electronic supplementary material, table S1 for results and statistics). Moreover, we found several cellular components that were over fourfold enriched with respect to the background, involving tRNA methyltransferase (GO:0043527, *p* < 0.01), endoplasmic reticulum Sec (GO:00312005, *p* < 0.01), translocon (GO:0071256, *p* < 0.05) and polysome (GO:0005844, *p* < 0.0001) complexes (electronic supplementary material, table S1). These complexes play key functions in post-transcriptional RNA modification, protein translocation into the endoplasmic reticulum and in the translation of mRNA into polypeptides [48–51]. Therefore, SL-type-1 appears to be involved in transcription and translation processes, and energy/metabolic management necessary for maintaining cellular function.

By contrast, *P. sinerae* SL-type-2 is enriched in processes regarding division and reproduction of the organism, such as DNA replication (GO:0006260, *p* < 0.05) and geometric change (GO:0032392, *p* < 0.01), the fission of organelles (GO:0048285, *p* < 0.001) and their inheritance to daughter cells (GO:0048308, *p* < 0.05), and in the moulting cycle (GO:0042303, *p* < 0.05, involved in the regeneration of the outer covering of the cell—figure 4*c*). This enrichment in biological processes is also in agreement with the enriched cellular components found for SL-type-2, where cell division site (GO:0032153, *p* < 0.001), chromosome (GO:0005694, *p* < 0.01) and cell wall (GO:0005618, *p* < 0.05) presented over a twofold enrichment compared to the background (electronic supplementary material, table S1). These contrasting patterns suggest the two different SL-types mark different transcriptome repertoires that are involved in discrete cellular functions.

In comparison, functional enrichment in *M. nigrum*, for which we only found evidence of a single SL type expressed within the zoospore transcriptome, demonstrates a clear overlap with both the two SL-types found in *P. sinerae*. Specifically, SL containing transcripts of *M. nigrum* zoospores are enriched for genetic transfer (GO:0009292, *p* < 0.05), organelle fission (*p* < 0.05), cytoplasm (GO:0007028, *p* < 0.05), organelle and peroxisome organization (*p* < 0.05) and DNA replication (*p* < 0.05) (figure 4*d*). These processes are related to the division and reproduction of cells to produce offspring, which overlaps with the enriched processes found in SL-type-2 of *P. sinerae*. Furthermore, the *M. nigrum* SL-bearing transcripts are also enriched for biological processes related to RNA processing (*p* < 0.05), transport of different molecules and metabolic activities (figure 4*d*), which overlap with functions of *P. sinerae* SL-type-1. Based on these data, we suggest that the further segregation of the *P. sinerae* transcriptome, based on two different SLs, was a recent innovation and that the two pools of transcripts with different SL-types were ancestrally encoded with a single SL-type, as is seen in *M. nigrum*.

Interestingly, all three subgroups of transcripts marked with an SL-type across the two species are functionally enriched for the biological process involved in ‘RNA trans-splicing via transesterification reactions' of nuclear mRNA via the spliceosome (GO:0000375, *p*-value < 0.05) (figure 4*b–d*; electronic supplementary material, table S1). Whereas in *M. nigrum* and *P. sinerae* SL-type-1, this function seems to be linked to the cellular components related to the ‘spliceosomal complex’ (GO:005681, *p*-value < 0.05) and the ‘small nuclear ribonucleoprotein complexes' (GO:0030532, *p*-value < 0.05), and are thus part of the integral structure of the spliceosome for pre-mRNA processing [52], these GO terms were not found enriched in the SL-type-2 (electronic supplementary material, table S1). Yet, several recovered *P. sinerae* SL-type-2 transcripts involved in ‘RNA trans-splicing via transesterification reactions’ are related to the spliceosome and snRNP complexes. Moreover, both SL-types *in P. sinerae* are enriched for the ‘exosome (RNase complex)’ (GO:0000178, *p*-value < 0.05), which have been reported to regulate RNA surveillance and turnover through the degradation of precursor mRNAs, controlling expression of the spliceosomal components and its assembly into the spliceosome [53]. These results suggest that the SL mark transcripts that encode components of the spliceosome and associated machinery, the very systems that catalyses their own function.

**Figure 5.**
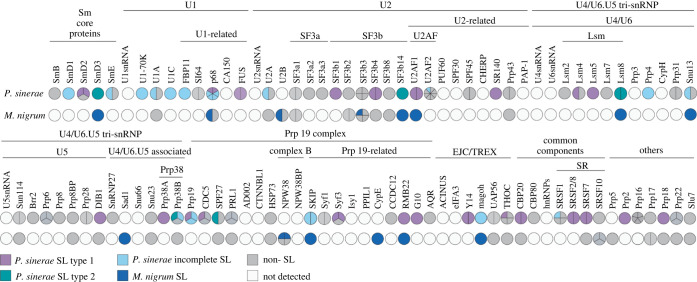
Spliceosome components encoded in SLe-bearing transcripts and transcripts without an SLe-sequence in *P. sinerae* and *M. nigrum* zoospores transcriptomes. Coulson plots [54] of genes encoding for spliceosome components in SL-bearing/lacking transcripts, identified from *P. sinerae* and *M. nigrum* transcriptomes. Each plot division represents the number of isoforms/paralogues encoding for that protein (see also electronic supplementary material, table S2). The different colours represent if they are encoded in transcripts/paralogues containing *P. sinerae* SL-type-1 (purple), *P. sinerae* SL-type-2 (green), *P. sinerae* incomplete SLe-sequences (light blue) or in transcripts without SLe-sequence (non-SL, grey). For *M. nigrum*, SL-bearing transcripts/paralogues are coloured in dark blue, and those without SL in grey. White circles indicate this protein has not been detected in the transcriptome dataset.

To investigate this further, we used KEGG annotation tools to identify all transcripts that encode candidate components of the spliceosome (see 'Methods'). This analysis demonstrates that numerous components of the spliceosome are encoded by transcripts which carry SLs (figure 5; electronic supplementary material, table S2). Interestingly, we can clearly see elements of *M. nigrum* SL-marked transcriptome differentially segregated between the *P. sinerae* SL-type-1 and SL-type-2 transcriptome (e.g. U2AF1 and RMB22 found in SL-type-1 and SMD3, SF3b14 and Lsm8 found in SL-type-2). Because both SL *trans*-splicing and genic *trans*-splicing share most of the basic machineries of the spliceosome, which carries out intron removal or *cis*-splicing [52], our results suggest that the association between SL-systems and spliceosomal-genes may be important for spliceosomal function and evolution, in agreement with the thought that that SL *trans*-splicing arose through evolution from *cis*-splicing or vice versa [2,55]. Specifically, the identification of such patterns may be important for understanding both the conservation of these systems (for example, why these systems are retained and possibly hard-wired into transcriptional function in some taxa) and the diversification of the pools of transcripts the SL system marks.

Overall, this study demonstrates a diversification of the SLTS-system in the Dinozoa lineage. This work also shows that diverse SL sequences are present across different Perkinsozoa taxa, and that this system diversified into multiple SL types encoded in parallel in *Parvilucifera sinerae*. That diversification seems to have led to a segregation of transcriptome based on the differential addition of variant SLe-sequences to transcripts encoding for proteins of different functional categories. Evidence of multiple SL types encoded in parallel in the same species has been reported in other eukaryotic lineages. The nematode *C. elegans* for example has two distinct types of SLs with different roles and is added to different sets of transcripts. *C. elegans* SL type-1 is incorporated at the start of the operon and SL type-2 is used to resolve the downstream coding sequences into different transcripts. The presence of SL1 in all nematodes studied suggests that it might have arisen in the ancestor of extant nematodes, whereas SL2 seems likely to have evolved from SL1 within the nematode radiation in the ancestor of the clade that includes *C. elegans* [56,57]. In order to understand the evolution and function of the SLTS-system in the Perkinsozoa, an effort should be made to sample a wide range of *Parvilucifera* species and related taxa, to identify at which point in the evolution of this lineage the use of multiple parallel SL sequences arose. Moreover, since Perkinsozoa parasites have complex life cycles, and currently there is no data pertinent to identifying changes in the SL system function across the life cycle, it will be of great interest to determine if the utilization of different SL sequences plays a different role in the variant transcriptional modules expressed during different stages of the *Parvilucifera* life cycle. To answer that question, we will need to apply new methods that will allow us to study this mechanism at the level of individual cells instead of ensemble transcriptome sequencing, allowing the identification of transcriptional characteristics associated with life cycle stages [58]. More widely, future work should also investigate the function of variant SLs encoded in parallel within the same cell across diverse taxa using methods that do not homogenize the SL sequence, the relationship between SL utilization and the transcript repertoire that encodes the spliceosomal apparatus, and the conservation of these systems across the tree of life. Such focus will be important for understanding the evolution of SL *trans*-splicing.

## 3. Conclusion

Here we used a range of genomic and transcriptomic data to investigate the diversification of the Perkinsozoa SLTS-system, showing diversification in the sequence and structure of the SLs and identifying how SLTS-system have evolved to mark different components of the transcriptome. In the case of *P. sinerae*, variants of the SLTS-system mark discrete functional categories of transcripts in parallel, effectively segregating the transcriptome into three pools. Our data also show that the SLTS-system marks transcripts with increased mRNA levels, suggesting that SLe's mark functional subgroups of transcripts for increased mRNA levels, either by increased mRNA production and/or protection of mRNA from degradation. All three SL-transcriptome pools investigated included genes that encode the spliceosome and associated machinery, suggesting that the spliced-leader system in Perkinsozoa drives increased representation of mRNA that encodes proteins important for its own function. We suggest that this mode, whereby the spliced-leader system is coupled to the transcripts that encode its own function, is important for understanding the evolution, retention and diversified uses of such systems.

## 4. Methods

### 4.1. Parasite and host culture and maintenance

Strains of host and parasites were isolated from the Catalan Coast (Mediterranean Sea) and were provided by the Institut de Ciències del Mar (ICM-CSIC), Barcelona, Spain. *Parvilucifera sinerae* (strain Arenys_2018) and *Maranthos nigrum* (strain Estartit_2017) cultures were maintained by transferring 2 ml of the parasite culture and 5 ml of *Alexandrium minutum* (strain Arenys_2008) at exponential growth phase to a 12-well plate (Sarstedt Inc), twice weekly. Dinoflagellate host cultures were grown in f/2 medium without silica and prepared using sterile artificial seawater [59]. All cultures were maintained at 20°C with a 12 h : 12 h light : dark photo-cycle with a photon flux of approximately 32 µmoles m^−2^ s^−1^.

### 4.2. Sample collection for nucleic acid extraction

To obtain cell samples of the zoospore stage of *P. sinerae* and *M. nigrum* parasitoids, 100 ml cultures of healthy *A. minutum* host, in 550 ml cell culture flasks with vented caps (Greiner Bio-One Ltd), were each infected by the addition of 50 ml of parasite culture. Individual co-cultures were then used for DNA or RNA sampling. Infected cultures were observed every day under an inverted microscope (Olympus CKX41) to the monitor progression of the infection. When most of the parasites completed the infection cycle and a high number of zoospores were observed swimming freely, two steps of sequential filtration were carried out in order to avoid contamination with DNA/RNA from the host or infected cells. The first step consisted of gentle filtration through a 5 µm polycarbonate filter (Whatman Nuclepore), allowing physical separation of the host (15–20 µm in size) from the parasite (2–4 µm in size). The pass-through fraction, which contained the parasite zoospores, was then filtered through a 0.8 µm polycarbonate filter to retain the zoospores. The filter was rinsed with sterile artificial seawater (autoclaved and 0.2 µm filtered) to remove residual bacteria.

To obtain samples for genomic DNA (gDNA) sequencing, filters were directly stored at −80°C before being processed. For RNA samples, zoospores were collected from the filter by scraping the cells into 1 ml LifeGuard Soil Preservation Solution (Qiagen). The zoospore-enriched solution were then centrifuged at 4°C for 10 min at 8000*g*, the supernatant removed, and the pellet resuspended in 350 µl of TRI reagent (Zymo Research). Samples were then stored at −80°C until RNA extraction.

We have focused transcriptome sequencing efforts on the zoospore phase of the life cycle because (i) we wanted to sample a single phase of the life cycle to minimize ensemble transcriptome signatures and (ii) the host-infected stages contained multiple phases of the life cycle some of which have proved recalcitrant to effective nucleotide sampling.

### 4.3. Nucleic acid extraction and library preparation

Total RNA was purified using a Direct-zol RNA Miniprep kit (Zymo Research) following the manufacturer's protocol. Briefly, tubes containing the samples in TRI reagent were thawed on ice, vortexed for 30 s and then centrifuged at 13 000*g* for 2 min. The supernatant was carefully transferred to a new tube without disturbing the cell debris pellet. RNA purification was completed including an in-column DNase treatment for 20 min. Total RNA was eluted in 30 µl of nuclease free-water, and 1 µl (2U) of RNasin Plus (Promega) was added to inhibit ribonuclease activity. Total RNA was quantified on a Qubit 4.0 Fluorometer using the Qubit RNA HS Assay (Thermo-Fisher Scientific). RNA quality was assessed on an Agilent 2200 TapeStation using the HS RNA screen tape assay (Agilent Technologies). Samples were stored at −80°C until library preparation and sequencing.

For all RNA samples, a 35-cycle PCR using general eukaryotic primers (TAReuk454FWD1 and TAReukREV3 [60]) was performed alongside a positive DNA sample control, following the PCR conditions in Stoeck *et al*. [60]. Agarose gel electrophoresis was performed to confirm the absence of DNA in the target samples. If PCR amplification was positive, a second DNase treatment was carried out using the TURBO DNA-free Kit (Invitrogen) and a PCR using the general eukaryotic primers and conditions described above was repeated to confirm the removal of DNA.

Three replicate culture and zoospore RNA extractions for *P. sinerae* and a single *M. nigrum* culture replicate were taken forward for sequencing. Samples of mRNA were then prepared for sequencing using a TruSeq DNA HT Kit (Illumina), including poly(A) RNA purification. Samples were sequenced using the MiSeq Illumina System (250 bp paired-end), with an extra sequencing run for the *M. nigrum* sample on a HiSeq System (250 bp paired-end). Library preparation and sequencing were performed at Exeter Sequencing Service (University of Exeter, Exeter, UK).

Genomic DNA was extracted using the AllPrep DNA/RNA Mini kit (Qiagen), following the manufacturer's protocol. Cells were harvested from the filter by scraping them into 350 µl of Buffer RLT containing β-mercaptoethanol. To homogenize the samples, cells were vortexed for 2 min with 400–625 µm acid-washed glass beads (Sigma Aldrich) and centrifuged for 1 min at full speed, and the homogenized lysate was transferred to an AllPrep DNA spin column. gDNA purification was completed following the manufacturer's protocol and DNA was eluted in 50 µl of Buffer EB. gDNA was quantified on a Qubit 4.0 Fluorometer using the Qubit dsDNA HS Assay (Thermo-Fisher Scientific). Quality of the DNA was assessed with the Agilent gDNA screen tape assay on an Agilent 2200 TapeStation system (Agilent Technologies). Whole-genome amplification was then carried out using the REPLI-g single cell kit (Qiagen) following the manufacturer's protocol. To obtain the maximum DNA yield, the reaction was incubated for 16 h. After stopping the reaction, the amplified DNA was immediately purified using Agencourt AMPure XP magnetic beads (Beckman Coulter). Amplified gDNA was quantified on a Qubit 4.0 Fluorometer using the Qubit dsDNA BR Assay (Thermo-Fisher Scientific). Quality of the amplified DNA was assessed, as above. Genomic libraries were prepared using Netflex Rapid DNA-Seq Kit (PerkinElmer) protocol and sequenced using the MiSeq Illumina platform (250 bp paired-end) by Exeter Sequencing Service (University of Exeter, Exeter, UK).

### 4.4. Severe Perkinsea Infection-associated tadpole meta-genomic nucleic acid extraction and library preparation

*Lithobates sylvaticus* tadpoles were collected from multiple sites in the Kenai Peninsula Borough of Alaska in June/July 2016 and processed by Smilansky *et al*. [61]. Tadpoles were confirmed to be infected by NAG01 Perkinsea by PCR amplification of a 290 bp fragment of the SSU rRNA gene using the primer pair 300F-B [25] and NAG01R_1 [61] using the PCR conditions and reaction mixture specified in Smilansky *et al*. [61], followed by sequencing of the PCR product (Eurofins Genomics).

Total DNA was extracted from two samples of liver tissue from SPI-positive tadpoles using the RNeasy PowerSoil DNA Elution Kit (Qiagen), according to the manufacturer's protocol. One gDNA sample contained nine different Perkinsea-infected individuals pooled together (KNA_DNA) at different Gosner stages, while the other sample contained the liver tissue of a single Perkinsea-infected tadpole (KNA03_01). Libraries were prepared using the Netflex Rapid DNA-Seq Kit (PerkinElmer). Sequencing was performed using the MiSeq Illumina platform (300 bp paired-end) by the Exeter Sequencing Service (University of Exeter, Exeter, UK).

All attempts to extract tractable RNA samples from the Alaska tadpoles and additional *Lithobates sphenocephala* tadpoles captured from central Florida in March 2019 failed.

### 4.5. Genomic DNA-sequence assembly

The genomes of *P. sinerae* and *M. nigrum* were assembled using the workflow script SAGAWE (https://github.com/guyleonard/sagawe/). Briefly, this automatically trims adaptors, performs quality controls, overlaps reads and then assembles the raw sequencing reads using Trim Galore v. 0.6.6, FastQC v. 0.11.9, bbmerge v. 38.9 and SPAdes v. 3.15.2 (bbnorm - digital normalization—was not used). Subsequently, a set of downstream assembly statistics were computed using QUAST [62], BUSCO [63] and BlobTools [64]. The assembled genome of *P. sinerae* resulted in 68 409 contigs with a total length of 40 152 151 bp and an N50 of 1847. BUSCO v. 5 genome analysis using eukaryota_odb10 and alveolata_odb10 databases reported Complete:23.5% [Single:23.5%, Duplicate:0.0%], Fragmented:20.0% and C:59.1% [S:58.5%, D:0.6%], F:8.2%, respectively. The assembled genome of *M. nigrum* resulted in 236 502 contigs with a total length of 81 661 540 bp and an N50 of 1380. BUSCO v. 5 genome analysis using eukaryota_odb10 and alveolata_odb10 reported C:22.7% [S:19.6%, D:3.1%], F:23.9%, M:53.4% and C:48.0% [S:40.4%, D:7.6%], F:13.5%, M:38.5%, respectively. Subsequently, the script ‘exonerate_protein_prediction.py’ from https://github.com/nickatirwin/Phylogenomic-analysis was run on the assembled genomes. This uses Exonerate v. 2.4.0 and the previously predicted proteins of the *Perkinsus marinus* (strain ATCC 50 983/TXsc) reference proteome (UP000007800; UniProt database)—the most closely related species from which the proteome with full genome representation is currently available—to produce a set of related protein predictions. These were then checked with BUSCO v. 5 to assess the completeness of the predicted proteins as a proxy for genome completeness. These *P. sinerae* and *M. nigrum* BUSCO v. 5 genome analyses using alveolata_odb10 reported C:53.8% [S:28.1%, D:25.7%], F:29.2%, M:17.0%, n:171 and C:46.8% [S:21.1%, D:25.7%], F:29.8%, M:23.4%, n:171, respectively. These data provide a partial genome sample sufficient for us to contrast gene and transcript structure, and to investigate splicing.

Using the same approach, we constructed genome assemblies for the two SPI-associated tadpole tissue samples. These data resulted in two assemblies, KNA_DNA and KNA03_01, with the following assembly statistics: KNA_DNA: total contigs 49 939; total length 49 692 219 bp; largest contig 17 524 bp; GC 42.55%; N50 1012 and KNA03_01: total contigs 54 199; total length 54 806 566 bp; largest contig 21 616 bp; GC 42.60%; N50 1,061. BUSCO analysis was not conducted as this is a ‘meta-genomic’ sample including frog DNA and a diverse associated microbiome.

### 4.6. Transcriptome assembly

Initially, raw paired-end Illumina reads were merged using PEAR v. 0.9.6 [65] and sequencing adapters were trimmed using fastp v. 0.20.1 [66]. Read quality was then assessed using FastQC v. 0.11.9 [67] before *de novo* transcriptome assembly was conducted using Trinity v. 2.12.0 [68]. In the case of *Parvilucifera*, three biological replicates (Z1, Z2 and Z3) were co-assembled together. In order to compare the three replicates, we performed transcript expression quantification using the program Salmon [69]. We generated a transcript counts matrix and performed cross-sample normalization using normalized expression values measured as transcripts per million (TPM). Then, transcripts were ordered by their abundance and the top 1000 most expressed transcripts in each replicate were selected, a total of 1134 unique transcripts. Overlap between the 1134 selected transcripts was visualized using the package *ComplexUpset* (v. 1.1.0), and their normalized expression values were plotted using the *heatmap.2* function implemented in *gplots* package (v. 3.1.3) in *R* studio (v. 1.1.463). For the comparison of the three replicates, see electronic supplementary material, figure S6. Proteins were then predicted from the resulting transcripts using TransDecoder v. 5.5.0 after comparing translated proteins to the SWISS-PROT database using Diamond BLASTp (sensitive mode, *E*-value < 10^−5^) [69–72]. The resulting assemblies and proteomes were then filtered for contamination to account for mixed culture conditions and sample cross-contamination. To this end, predicted proteins were compared against each other, dinoflagellate transcriptomes (*Alexandrium catenella* MMETSP0790 and *Prorocentrum cordatum* MMETSP0055), and fungal and prokaryotic UniProt reference proteomes using Diamond BLASTp (sensitive mode, *E*-value < 10^−5^, query coverage >50%) [70,73,74]. Hits were then filtered by 99% (for Perkinsozoa transcriptome comparisons), 85% (for dinoflagellate and fungal hits) and 50% (for prokaryotic hits) identity, and the remaining matches were subsequently removed from the initial datasets. The ‘completeness’ of the filtered dataset was assessed using BUSCO v. 5 and comparisons with the alveolata_odb10 database.

### 4.7. Identification of the spliced-leader exon sequences in transcripts of Perkinsea infecting dinoflagellates

The three *P. sinerae* zoospore transcriptome sequencing library replicates (Z1, Z2, Z3) were co-assembled using the program RNA SPAdes v. 3.12 [75] without adaptor trimming and using default settings. This allowed for the transcripts to be assembled with the Illumina adaptor sequences intact. Ordinarily adaptors would be trimmed from the raw reads; however, as the SLe is located at the 5′ end of a transcript, part of the 5′ may be removed by the trimming process, thus making it hard to detect full-length 5′ modifications. Taking a random sample of 1000 assembled transcripts, we were able to visually inspect the assembled transcripts (using SeaView v. 4.7 [76]), identify the Illumina adapter at the 5′, align these bases across the transcripts and confidently remove the Illumina adapter. This process clarified the remaining 5′ end of the transcript sequence, allowing us to identify an identical short sequence across numerous different transcripts, a putative SLe. The same approach was conducted for the *M. nigrum* transcriptome library.

Once candidate SLe-sequences were identified, transcripts containing an SLe at the 5′ end were retrieved from the transcriptome. To this end, transcripts containing the highly conserved SL motif ‘CTTGAGCC’ within 100 nucleotides of their 5′ end were extracted. SLe-sequences were trimmed after the 3′ acceptor splice site (AG) immediately downstream of the SLe-sequence. All SLe were aligned using ClustalO [77], and the consensus sequence (threshold of 60%) was obtained using Seaview v. 4.7 [76]. Notably, missing information at the 5′ end of SLe-sequences is expected in RNA-seq data due to incomplete and low coverage sequencing of transcript ends [29]. Whenever there was apparent sequence data missing for multiple 5′ end positions in a sequence for a given transcript type, the conservation of these positions was measured considering only the recovered 5′ end sequence. To better observe variability within the SLe-sequences in each species, alignments of SLe for each species were used as input for the generation of ‘sequence logos’ using the WebLogo suite [27].

Next, we noted the presence of sections of sequence upstream from the canonical SL in *M. nigrum* and *P. sinerae* mRNA which showed no conservation (for example, electronic supplementary material, figure S7). For this reason, the consensus sequence was then used to retrieve SLe from genomic data using a BLASTn-short search at 90% coverage, allowing us to verify the canonical sequence for each species and detect the presence of different SLe types (as observed in *Parvilucifera*). To verify that those sequences were *trans*-spliced to different transcripts post-transcriptionally, annotated transcripts containing the SLe were BLAST searched (BLASTn) against the correspondent genome assembly (per cent identity (PID) greater than 95%). The 5′ ends of transcripts and their corresponding genomic sequence were aligned using Seaview v. 4.7 [76] to identify the 5′ UTR containing the SLe, the 3′ acceptor splice site (AG), the starting codon (ATG) and the coding sequence (CDS) (electronic supplementary material, figure S2).

Once the candidate SL sequences were confirmed as SLe *trans*-spliced post-transcriptionally onto mRNA, transcripts were then classified as possessing either a SL-type 1 or SL-type 2 based on the sequence characteristic at nucleotide position 13 and 14 (figure 1*b*) in the *Parvilucifera* SL sequence. In transcripts where the SL sequence was partial and the nucleotides that were missing included this variable region, the SL sequence could not be classified as either SL-type 1 or SL-type 2, and thus, it was classified as transcript with ‘incomplete SL’. Since *M. nigrum* possessed only one detectable SL sequence type, when partial SL sequences were detected at the 5′ end of transcripts, they were classified as SL-containing transcripts. The rest of transcripts with no detectable SLe at the 5′ end includes both those transcripts that genuinely do not have an SL because they are not *trans*-spliced and transcripts where the entire SL sequence is missing (partial 5′ ends) due to incomplete sequence sampling; these were classified as non-SL transcripts.

To assess the relationship between the SLe-sequences in Dinozoa clade, we aligned the consensus SLe-sequences obtained in the present study with those of dinoflagellates, *Amoebophrya* and *Perkinsus* reported in previous studies [6,9,11,12,28] allowing for gaps. A parsimony phylogenetic tree was generated using gaps as a 5th character state using the tree building tools available in Seaview v. 4.7 [76].

### 4.8. Spliced-leader PCR-based library, sequencing and assembly

*P. sinerae* and *M. nigrum* culture samples containing a mix of life cycle stages were used for SL-based library preparation. Parasitoid cultures were prepared by adding 50 ml of the parasitoid to 100 ml of host culture growing exponentially in 550 ml cell culture flasks with vented caps (Greiner Bio-One Ltd). Subsamples of 30 ml were collected at 24 h, 48 h, 72 h and 96 h after initial parasitoid inoculation. Each subsample was gently filtered through a 0.8 µm polycarbonate filter (Whatman Nuclepore) using a vacuum pump, in order to retain all infected cells in the filter. After filtration, cells were harvested from filters, centrifuged and stored as explained in §4.2. Before RNA extraction, the four subsamples of each parasitoid species taken at a different stage of the culture were pooled together in order to obtain one sample containing all parasite life cycle stages. Total RNA was extracted as explained in §4.3. Approximately 500 ng of total RNA was used as a template to synthesize the first-strand cDNA with Superscript III Reverse transcriptase (Life technologies), using a GeneRacer Oligo dT Primer (Invitrogen), and following the manufacturer's protocol. First-strand cDNAs were treated with 1 µl (2 µ) of Ribonuclease H (New England Biolabs Inc.) and used as a PCR template. Prior to PCR amplification, cDNA was quantified on a Qubit 4.0 Fluorometer using the Qubit ssDNA Assay kit (Thermo-Fisher Scientific). 10 ng of total cDNA was used as a starting material for PCR amplification and PCRs using two different SL-primers pairs for *P. sinerae* and one primer pair for *M. nigrum* were carried out in triplicate.

In order to amplify full-length transcripts containing the splice-leader at the 5′ end, PCRs were carried out using the GeneRacer 3′ Primer as a reverse primer (Invitrogen) paired with forward primers corresponding to the splice-leader sequences identified from the transcriptome and genome datasets (electronic supplementary material, table S3). GoTaq G2 Hot Start Green Master Mix (Promega) was used and PCR was performed using the following cycling conditions: 95°C for 2 min followed by 32 cycles of 95°C for 30 s, 54–67°C for 40 s, 72°C for 2 min and a final extension at 72°C for 10 min. The annealing temperature for each primer pairs was initially estimated using the Tm calculator (ThermoFisher scientific). A gradient PCR based on the estimated temperature was used to determine the optimal annealing temperature for each primer pair (electronic supplementary material, table S3). The 50 µl reaction mix contained 1X of the PCR Master mix, 1500 nM GeneRacer 3′ Primer, 500 nM splice-leader forward primer and 2 µl of template (approx. 10 ng). All cDNA amplicons were checked by gel electrophoresis on a 1.5% agarose gel, and the PCR product was purified using the Wizard SV Gel and PCR Clean-Up System (Promega).

Libraries were prepared using the NEXTFLEX Rapid DNA-Seq Kit (PerkinElmer) without cDNA fragmentation in order to get both transcripts ends. Sequencing was performed on a MiSeq Illumina System (250 bp paired-end) at the Exeter Sequencing Service (University of Exeter, Exeter, UK).

We removed low-quality reads and performed Illumina adaptor trimming using Trim Galore v. 0.6.6 with default options. Primers and polyA tails were then trimmed using cutadapt v. 4.1 and read quality was assessed using FastQC v. 0.11.9 [60] before de novo transcriptome assembly was conducted using Trinity v. 2.12.0 [61]. Because we did not fragment the cDNA before sequencing, the assembly was performed considering the reads as unpaired.

### 4.9. Comparative analysis between illumina RNA-seq and spliced-leader PCR-based libraries

In order to experimentally test whether the transcripts without a detectable SL were transcripts that genuinely are not *trans*-spliced or are missing the SL due to technical limitations of the RNA-seq sequence sampling methods [29], we compared the transcripts from the spliced-leader multiplex PCR-based libraries with the different pools of transcripts classified as SL-bearing transcripts or without from the Illumina RNA-seq libraries using BLAT [78]. Hits were filtered by a minimum percentage of identity 98% and a minimum alignment length of 50 nucleotides to avoid random hits from very short-sequence alignments. Finally, hits were sorted by unique sequence IDs. Shared transcripts between the different libraries were visualized using *VennDiagram* R package (v. 1.7.0) in R studio (v. 1.1.463).

### 4.10. Identification of the spliced-leader-RNA ORFs from genome assemblies

The consensus SLe-sequences identified in the 5′ UTR of *P. sinerae* and the *M. nigrum* transcripts were used to query each parasite genome to identify SL-RNA genes using BLASTn-short-sequence similarity searches. Because we only have transcriptome data from the free-living zoospore stage and these parasites have several life cycle stages, hits to the genome with greater than 95% sequence identity and query coverage 100% were collected and aligned using Seaview v. 4.7 [76], in case different SLe-variants from the ones detected in the transcriptomic data were present. Sequences were manually curated, and candidates containing the complete SLe-sequences followed by an intronic region of a minimum of 40 nt were selected. Only those sequences where the 5′ splice donor site (GU) was followed downstream by a poly-T tract were selected as potential SL-RNA ORFs. When more than one SL RNA sequence was retrieved for a given species, ClustalO [77] alignments were performed in Seaview v. 4.7 [76] to guide redundancy reduction (such as removal of completely identical sequences) and false positive discovery. In the case of Perkinsea infectious agent of tadpoles, due to the absence of transcriptomic data, a BLASTn-short (PID 90%) search using the SLe-sequences identified from a range of Dinozoa [6,9,11,12] was performed against the two genomic assemblies (KNA03_01 and KNA_DNA) to extract putative SL-RNA genes.

### 4.11. Generation of spliced-leader-RNA gene secondary structure

To analyse structural conservation and topological features of the potential SL-RNA gene in Dinozoa, all SL-RNA exon/intron ORF sequences were submitted to Mfold online service [32] with the folding temperature set at 20°C (the temperature at which the parasite cultures were maintained) for RNA structure generation and energy assessment. In the case of *K. brevis*, *Amoebophrya* and *Perkinsus* sequences obtained from previous studies; folding temperatures were set at 20°C, 15°C and 27°C, respectively (temperatures at which these organisms were maintained in culture) [6,9,11,12,28]. Structures were then visually analysed for comparison.

### 4.12. Phylogenetic analysis of small subunit and large subunit ribosomal genes in Dinozoa

SSU and LSU rRNA sequences from the transcriptome sequence assembly of *P. sinerae* and *M. nigrum* were retrieved using BLASTn searches using the following GenBank sequences as search seeds: KM878667, KM878666, MN721813 and MN721817. For the parasite of tadpoles, the SSU rRNA sequence from the *Rana sphenocephala* pathogen available in GenBank database (accession number EF675619) was used as a search query to retrieve SSU sequence from the meta-genomic sequence assembly. Since no LSU sequences are publicly available for the NAG01 clade, the *Perkinsus marinus* LSU sequence (accession no. EU919450) was used as query to obtain the parasite LSU sequence from the genome. SSU and LSU rRNA sequences mentioned above and sequences belonging to six dinoflagellate species (accession no.: AF099183/AY571373, JF715165/JX402086, KF925338/AY571372, AY121846/AY245693, EF492502/AY355458 and JN986577/DQ114466), one Syndiniales (AF069516/HM483395) and two *Perkinsus* species (AF324218/EU919450, AF102171/MF186934), from which SL-RNA gene was previously identified, were used to reconstruct the phylogenetic tree. The concatenated SSU and LSU rDNA-sequences were aligned using MAFFT v. 7.2 [79], using the automatic option, and the alignment was manually checked using Seaview v. 4.7 resulting in 1757 positions for SSU sequences and 1118 positions for LSU sequences. Subsequently, the alignments were trimmed with TrimAL [80] using automated trimming heuristic (automated1) option resulting in an alignment of 2293 positions (1625 for the SSU and 668 for the LSU). The maximum-likelihood phylogenetic tree was inferred with IQ-TREE v. 2.0 [81] under GTR + F + I + G4 and TN + F + R3 models for SSU and LSU genes, respectively, using edge-linked partition model [82] as selected by ModelFinder [83] based on the Akaike information criterion. We obtained node and branch supports with SH-aLRT test [84] and ultrafast bootstrap (UFBoot) with 1000 replicates [85] implemented in the IQ-TREE software. Trees were visualized with iTOL (https://itol.embl.de/).

### 4.13. Protein annotation and gene ontology enrichments

To examine the function of proteins encoded by SL-transcripts, proteins predicted from the transcriptomes were annotated using PANNZER2, eggNOG-mapper v. 2.1.2 and InterProScan v. 5.51–85 using the Pfam and PANTHER databases [86–91]. The resulting GO-terms were then simplified by mapping the terms to the Protein Information Resource GO-slim subset using Map2Slim (https://github.com/owlcollab/owltools/wiki/Map2Slim). GO enrichment was assessed by comparing the frequency of individual terms among transcripts with a given SLe-sequence (i.e. the test-set) to the transcriptome as a whole (i.e. the background). Significance was assessed using permutation tests with test distributions produced by generating 100 000 randomized samples, equal in size to the test-set, by randomly selecting transcripts from the transcriptome without replacement. Enriched GO-terms (*p* < 0.05) were summarized and visualized using REVIGO [92]. Annotated transcripts containing the different SLe were blasted (BLASTn) against each other using PID greater than 95% and query coverage greater than 95% for *P. sinerae* SL-type 1 versus SL-type 2 transcripts, and PID greater than 60% and query coverage greater than 50% for comparison between *P. sinerae* transcripts and SL-transcripts of *M. nigrum*. Shared transcripts between the different SLs were visualized using the *VennDiagram* R package (v. 1.7.0) in R studio (v. 1.1.463).

### 4.14. Mapping frequency analysis of relative transcript abundance patterns of transcripts with spliced-leader exon sequences and transcripts without

TPM read normalization and quantification was calculated for each *P. sinerae* RNA-seq library (Z1, Z2 and Z3) generating different matrices. The same process was performed for the two *M. nigrum* datasets (Miseq and Hiseq). Subsequently, for each species, we joined the tables generated into one by the ‘name and TPM’ columns and made sum and mean values of the TPM columns. Finally, we read in the lists of transcript versus identified SL-type-1, SL-type-2 and non-SL transcripts for *P. sinerae* and SL and non-SL transcripts for *M. nigrum*, and unified them to one column in the main table. The different datasets were plotted as ‘raincloud’ style plots (halved violin, plus a boxplot, plus scattered raw data) removing the first quartile in order to remove low read coverage transcripts which may falsely under-sample 5′ transcript regions. To assess statistical differences between the different datasets of *P. sinerae* three-way comparison we used the Kruskal–Wallis one-way ANOVA, and for the two groups in *M. nigrum*, we used the Mann–Whitney *U*-test using the ‘ggstatsplot’ R package [93]. We also ran significance tests between each group with the Wilcox test.

### 4.15. Identification of transcripts encoding spliceosome components

For *P. sinerae* and *M. nigrum* transcriptomes, KEGG annotations were recovered using eggNOG-mapper v. 2.1.2. These transcripts were previously sorted into bins identified by the presence of a particular SLe, an incomplete SLe or the absence of a SLe. These transcripts were then split into lists containing their transcript ID and related KEGG annotations for each individual bin. We then used the ‘KEGG Mapper – Reconstruct’ [94] online webserver (https://www.genome.jp/kegg/mapper/reconstruct.html) to generate KEGG pathway maps for each bin. We then downloaded the maps for Spliceosome annotation to identify which components were present in each bin. Further, the KEGG identifier and IDs of the transcripts present in the spliceosome for each SL-type and non-SL were recovered and the respective amino acid sequences were sorted into individual fasta files. KEGG identifiers were manually searched in the KEGG website to identify the specific spliceosome components encoded in each transcript and to count the number of paralogues present in order to generate Coulson plots.

## Data Availability

Sequencing data generated in this study have been deposited at the NCBI Sequence Read Archive (BioProject: PRJNA798887; BioSample: SAMN25131783 and SAMN25131784; and BioProject: PRJNA800762; BioSample: SAMN25280664 and SAMN25280665). The data are provided in the electronic supplementary material [95].
